# Complete Response After Pre-Operative Transcatheter Arterial Chemoembolization for Unresectable Primary Hepatic Neuroendocrine Tumour: A Case Report and Literature Review

**DOI:** 10.3389/fonc.2022.893403

**Published:** 2022-05-25

**Authors:** Xinyuan Zhang, Huiru Yang, Yujing Xin, Yi Yang, Haizhen Lu, Xiang Zhou

**Affiliations:** ^1^Department of Interventional Therapy, National Cancer Centre/National Clinical Research Centre for Cancer/Cancer Hospital, Chinese Academy of Medical Sciences and Peking Union Medical College, Beijing, China; ^2^Department of Pathology, National Cancer Centre/National Clinical Research Centre for Cancer/Cancer Hospital, Chinese Academy of Medical Sciences and Peking Union Medical College, Beijing, China

**Keywords:** primary hepatic neuroendocrine tumor, treatment, transcatheter arterial chemoembolization, complete response, case report

## Abstract

**Background:**

Primary hepatic neuroendocrine tumours (PHNET) are extremely rare. Currently, no evidence-based guidelines are available for PHNET treatment, especially for unresectable tumours.

**Case Presentation:**

We present the case of a 43-year-old man who was admitted to our hospital with complaints of backache for more than 1 month. The imaging examination showed a 5.5×5.3 cm lesion in the liver and no extrahepatic lesions, which was confirmed as a grade 2 PHNET by the pathological results and exclusion of non-hepatic origins. A multidisciplinary team (MDT) consultation revealed that the lesion was an unresectable primary hepatic neuroendocrine tumour (uPHNET) but could be potentially treated by conversion surgery. The patient was initially administered four cycles of chemotherapy with temozolomide, 5-fluorouracil, and ondansetron, and was evaluated as stable disease (SD) according to the Response Evaluation Criteria in Solid Tumours version 1.1 (RECIST 1.1). Because of the limited clinical benefit of chemotherapy, the patient subsequently underwent transcatheter arterial chemoembolisation (TACE) treatment, which reduced the tumour size and converted uPHNET to resectable tumours. A complete response (CR) was achieved after surgery, and the patient has been disease-free.

**Conclusions:**

This case was reported by a patient with uPHNET who benefited from the pre-operative TACE, providing a potentially effective management strategy for refractory tumours.

## Introduction

Neuroendocrine tumours (NETs) are rare tumours that originate from the diffuse neuroendocrine cell system, most commonly in the gastrointestinal tract, followed by the lung, and usually metastasize to the liver (1, 2). Primary hepatic neuroendocrine tumours (PHNET) are extremely rare neuroendocrine tumours, with less than 150 cases reported in the literature (3). The clinical manifestations of PHNETs lack specificity, and very few patients present with symptoms of carcinoid syndrome (4). Hence, PHNETs often remain undetected until the late stages of the disease. Moreover, the imaging performance of PHNETs is not specific, and it is difficult to distinguish radiologically from other liver carcinomas, such as intrahepatic metastatic neuroendocrine tumour, hepatocellular carcinoma (HCC), and intrahepatic cholangiocarcinoma (ICC) (3, 5). The definite PHNET diagnosis principally results from pathological examination and exclusion of other primary tumour sites, which requires extensive preoperative examination and evaluation and long-term follow-up to search for extrahepatic primary tumours (3, 4, 6). Percutaneous liver biopsy may be beneficial for preoperative diagnosis (6). To date, no therapeutic guidelines for PHNETs have been established, but surgical resection is thought to be the most effective treatment (7, 8). In the case of unresectable PHNET (uPHNET), the optimal treatment modality remains debatable. Transcatheter arterial chemoembolisation (TACE), liver transplantation, and chemotherapy have recently emerged as potential treatment options (8–11). In this study, we report a complete response (CR) of uPHNET according to the Response Evaluation Criteria in Solid Tumours version 1.1 (RECIST 1.1) with conversion surgery to achieve r0 resection after TACE, an outcome that has not been reported previously.

## Case Description

A 43-year-old man visited our hospital in May 2019, presenting with backache for over 1 month. The relevant medical history included a smoking history of 20 packs/years. No alcohol consumption or history of diabetes or hepatitis. A physical examination revealed no abnormalities.

**Figure 1:**
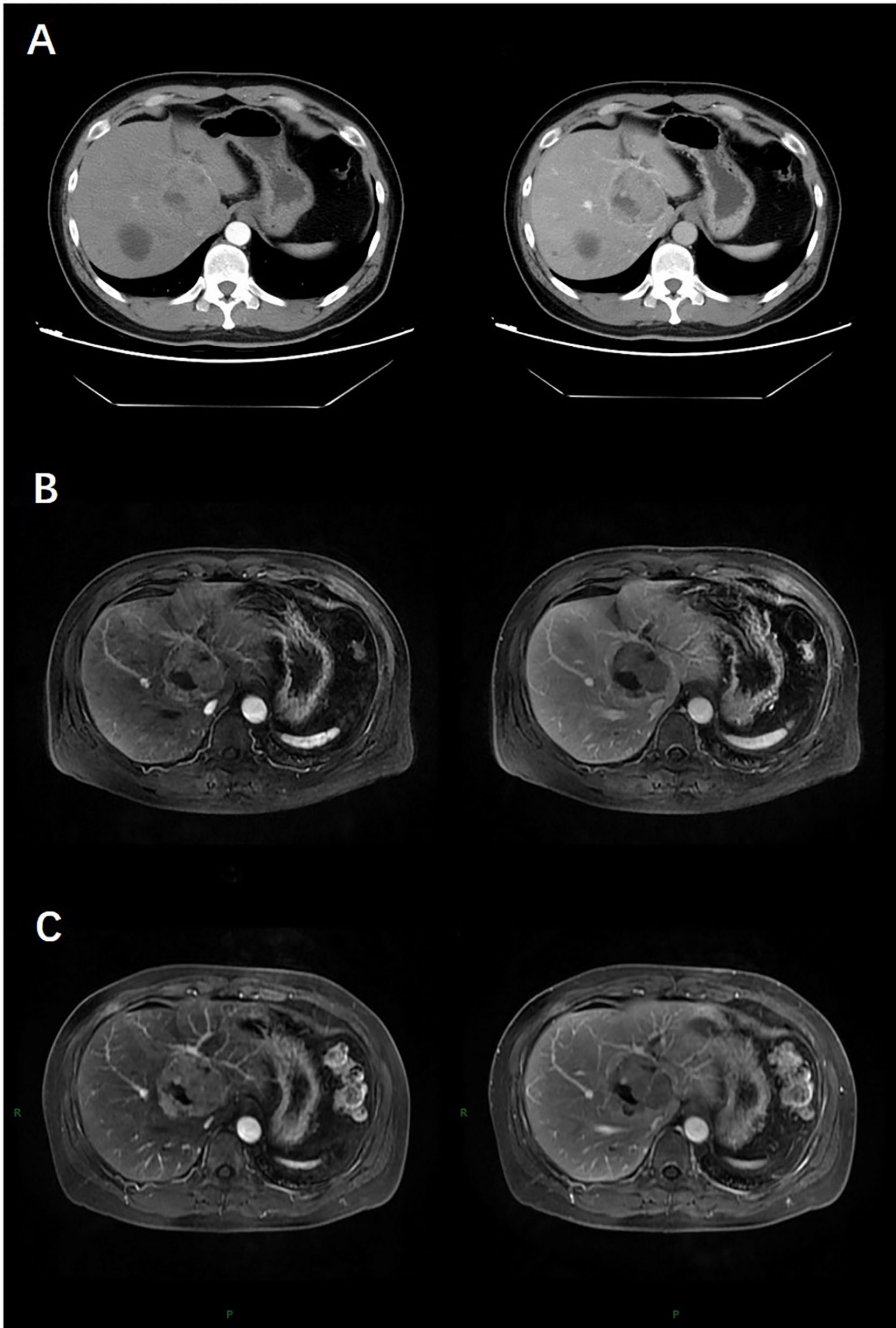
At baseline (May 2019), contrast-enhanced CT **(A)** and MRI **(B)** indicated a 5.5-cm lesion in the liver with obvious enhancement on arterial phase images and washout on delayed images. After four cycles of chemotherapy (July 2019), contrast-enhanced MR **(C)** indicated stable disease (SD).

The patient underwent abdominal contrast-enhanced computed tomography (CT) scan. The CT showed a 5.5×5.3 cm solid mass in the liver with a close relationship to the left portal vein and had no extrahepatic lesions (**Figure 1A**). No abnormalities were identified in other abdominal organs such as the pancreas, spleen, kidneys, and adrenal glands. Subsequent magnetic resonance imaging (MRI) revealed a 5.0 cm-sized well-defined solid mass with a cystic component, which was adjacent to the middle hepatic vein and left portal vein with peripheral biliary ductal dilatation and compression of the left branch of the portal vein (**Figure 1B**). The solid mass showed arterial hyperenhancement and washout on delayed imaging, strongly indicating HCC.

His preoperative laboratory data were as follows: direct bilirubin (DBIL) 6.0 umol/L (normal range 0.00–5.10), indirect bilirubin (IBIL) 13.0 umol/L (normal range 0.0–11.97), triglyceride (TG) 2.57 mmol/L (normal range 0.45-1.69), high-density lipoprotein cholesterol (HDL-CHO) 0.86 mmol/L (normal range 0.90-1.45), and low-density lipoprotein cholesterol (LDL-CHO) 3.62 mmol/L (normal range<3.34). Serum levels of tumour markers such as alpha-fetoprotein (AFP) and carcinoembryonic antigen (CEA) were within normal ranges.

The patient underwent oesophagogastroduodenoscopy and chest CT examination to identify extrahepatic lesions, which revealed normal findings. Because of the lack of obvious characteristics of the preoperative diagnosis, the patient underwent an ultrasound-guided core biopsy needle of the liver lesion 1 week after admission. Microscopic examination of the biopsy specimen (diameter: 0.3 cm) from the intrahepatic mass revealed tumour cells with abundant eosinophilic cytoplasm and uniform round nuclei. Immunohistochemical staining revealed that the tumour cells were positive for cytokeratin AE1/AE3, cytokeratin 18, synaptophysin (Syno), and chromogranin A (ChrA). Ki67 index was approximately 5%. A final diagnosis of NET (grade 2) was established.

We recommended a PET-CT scan for systemic evaluation to rule out metastatic neuroendocrine carcinoma. One month later, the patient underwent somatostatin receptor scintigraphy (gallium 68 PET-CT scanning) at an outside hospital (Peking Union Medical College Hospital, Beijing, China). The results revealed high somatostatin expression inside the liver, and no abnormalities were found outside the liver, confirming the PHNET diagnosis.

Considering the extent of the tumour lesions in both the right and left hepatic lobes, as well as the left portal vein, a multidisciplinary team (MDT) consultation showed that the lesion was uPHNET but could be potentially treated by conversion surgery.

To downsize tumours and convert uPHNET to resectable tumours, the patient was initially administered four cycles of chemotherapy with temozolomide, 5-fluorouracil, and ondansetron on July 16, 2019. MRI re-examination was performed after chemotherapy on October 9, 2019, revealing no change in the tumour size, which was evaluated as stable disease (SD) according to RECIST 1.1 (12) (**Figure 1C**).

Because of the limited clinical benefit, we decided to administer TACE with lipiodol embolism (5 ml) and suitable polyvinyl alcohol particles. The procedure was performed by an experienced vascular surgeon. After surgery, the CT results showed visible lipiodol deposition and necrosis of a few tumour masses, but some active lesions remained. TACE was possibly beneficial for patients in clinical evaluation. Therefore, the patient subsequently received three TACE treatments with lipiodol embolism (8 ml) and suitable polyvinyl alcohol particles for the same tumour lesion after the first TACE. CT re-examination or MRI results revealed further reduction in tumour size and marked tumour necrosis. Partial response (PR) status was evaluated according to the RECIST 1.1 criteria. Meanwhile, liver function and tumour marker levels were within normal ranges during the treatment and follow-up periods.

**Figure 2:**
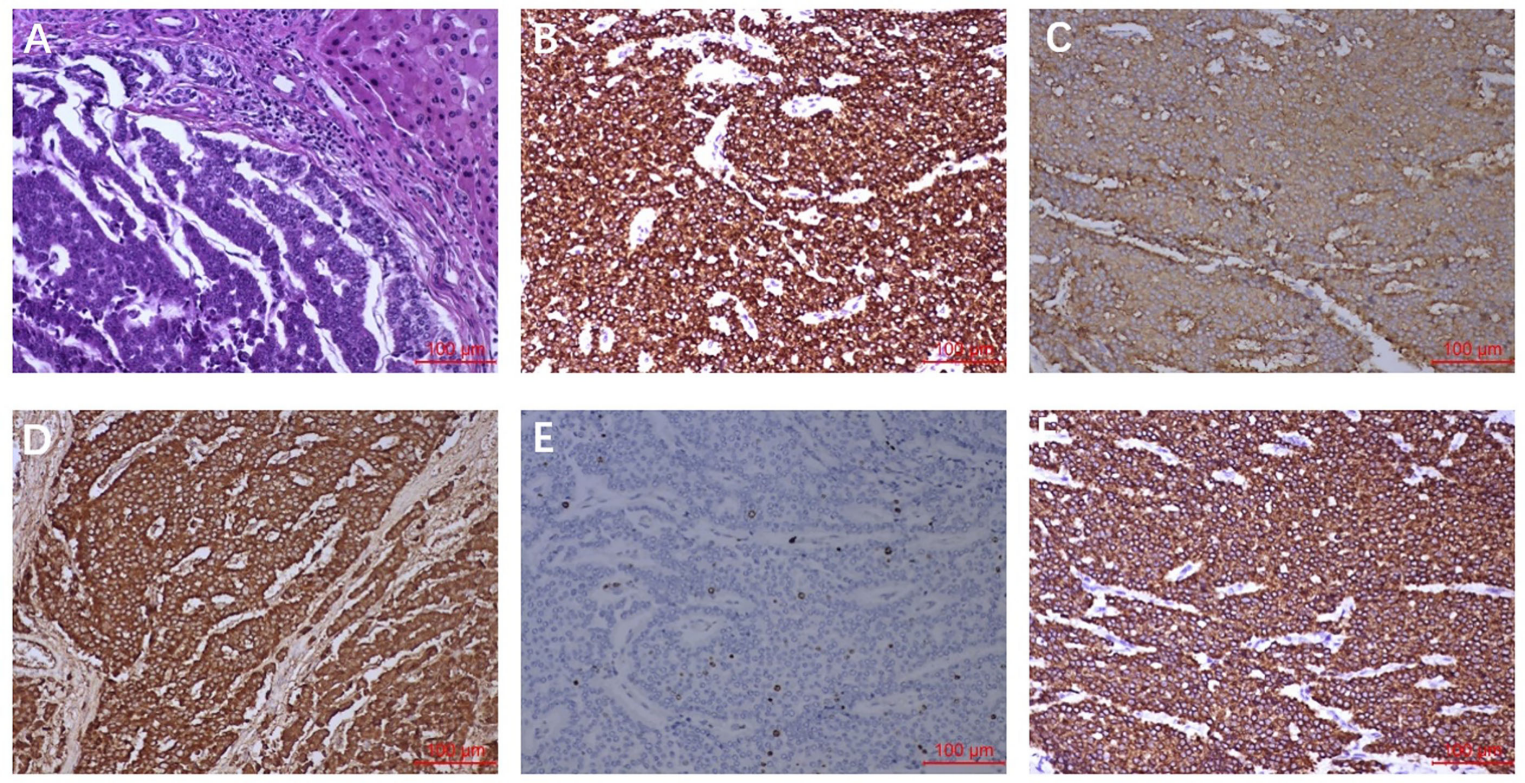
Pathological findings. **(A)** HE staining showed the tumour cells were arranged in nest and mass cord shape, and the cells were small and consistent. **(B)** AE1/AE3 staining showed diffuse cytoplasm was strongly positive. **(C)** Syno staining showed diffuse cytoplasmic positive. **(D)** ChrA staining showed diffuse cytoplasmic positive. **(E)** Ki-67 staining showed nuclear positive was about 5%. **(F)** CK18 staining showed diffuse cytoplasmic positive.

**Figure 3:**
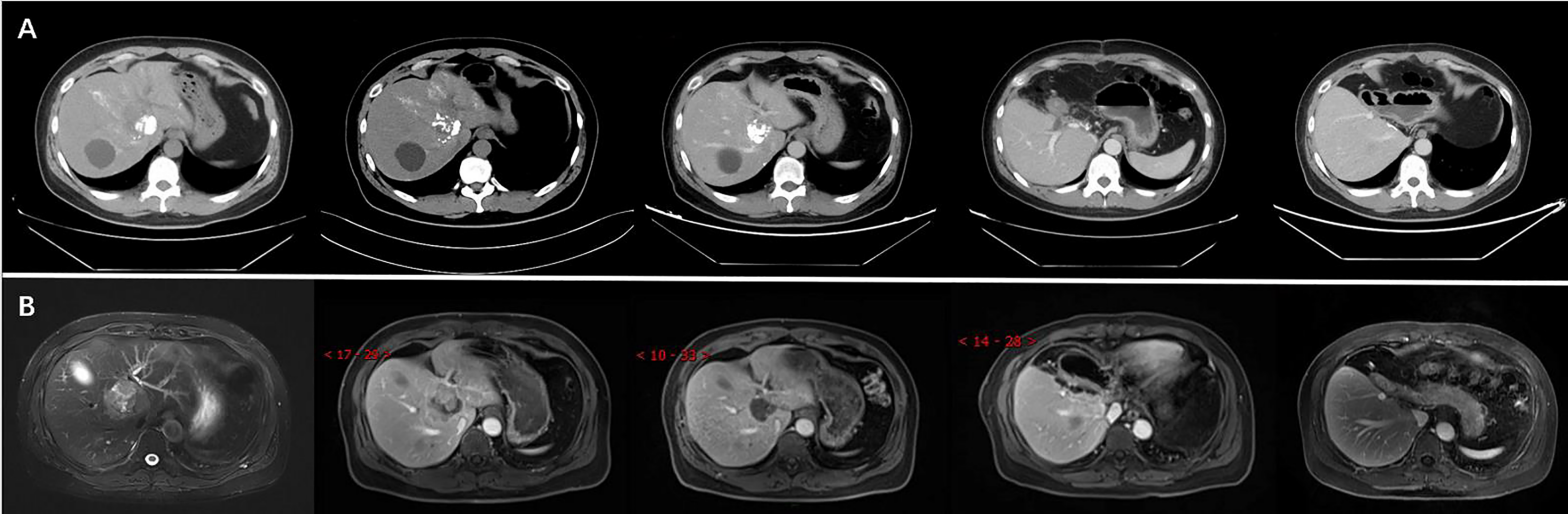
The CT **(A)** and MRI **(B)** revealed reduction of tumour size after TACE and confirmation of complete response (CR) after surgery.

Imaging results showed that the lesion had shrunk to an ideal size for surgery. Approximately 1 month later (September 16, 2020), the patient underwent left hepatic lobectomy, caudate lobectomy, and cholecystectomy and had an uneventful postoperative course. The resected specimen revealed a solid tumour measuring 3.2 cm × 3.0 × 2.8 cm, and the cut surface of the tumour was gray-yellow in colour with large regions of necrosis. Immunohistochemical staining revealed that the tumour cells were positive for AE1/AE3, CK18, Syno, and ChrA, with a Ki67 index of 5% (**Figure 2**). After treatment, the patient was regularly followed up, and each 3-monthly CT confirmed CR, exhibiting no signs of tumour progression (**Figure 3**). No abnormalities were observed in any of the laboratory examinations. Moreover, the possibility of a metastatic tumour from an extrahepatic primary site was ruled out by radiographic examination during the follow-up.

**Figure 4:**
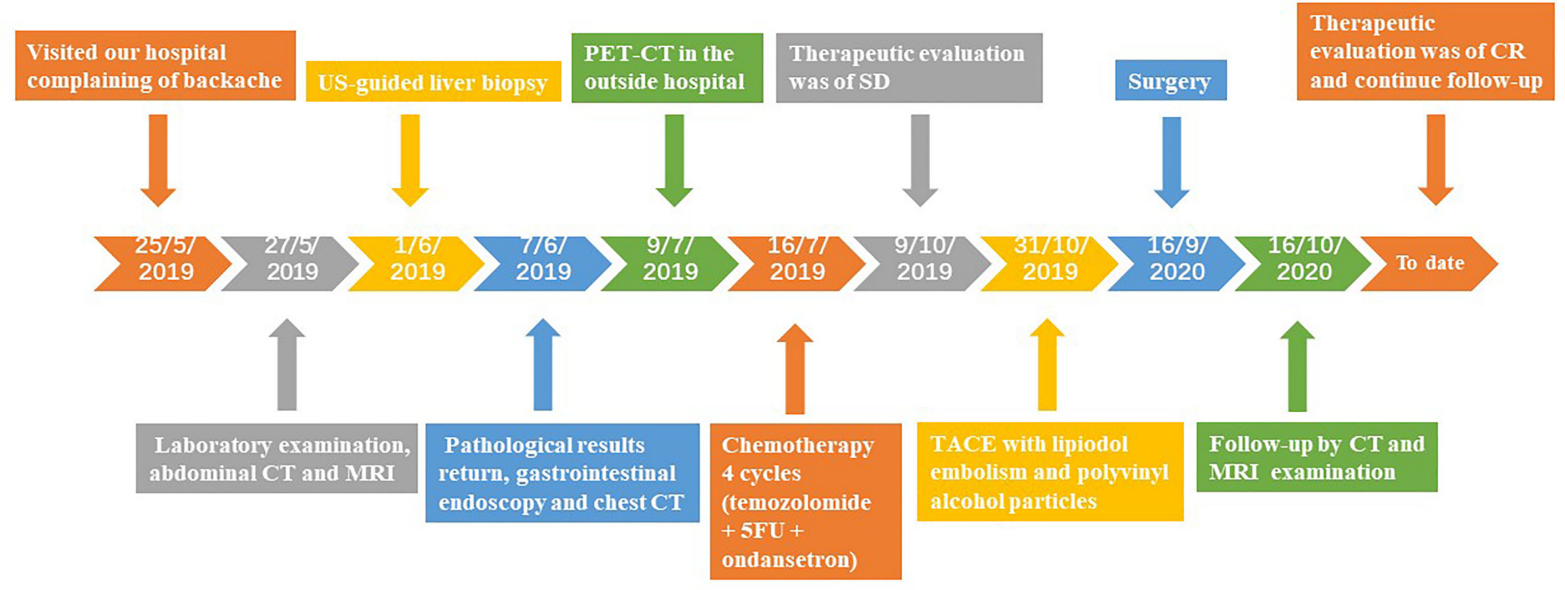
Timeline of the patient’s diagnosis and treatments.

The patient was in CR at the 15-month follow-up and remained disease-free. The timeline of the patient is summarised in **Figure 4**. Written informed consent was obtained from the patient for the publication of potentially identifiable images or data included in this article.

## Discussion

We presented a rare case of a middle-aged man with uPHNET that was successfully treated with a combination of pre-operative TACE and surgery. Our findings indicate that TACE could benefit the conversion treatment of uPHNET, prolonging disease-free survival in patients with limited clinical benefit from chemotherapy.

Previous publications have shown that PHNET is extremely rare (0.38%) among all liver tumours (13). Due to its rarity, it is challenging to diagnose PHNET before pathologic evaluation of a resected specimen by liver biopsy or surgery. PHNET usually presents with silent manifestations, and only 6.8% of patients present with symptoms of carcinoid syndrome, such as diarrhoea, abdominal pain, and skin flushing (3, 4, 6). In our case, the patient presented with a backache for 1 month without typical carcinoid syndrome.

PHNET is extremely difficult to diagnose precisely from imaging studies which display slight or significant arterial enhancement of the tumour parenchyma in the arterial phase followed by washout in the portal and/or late phases (14). Some cases have revealed that large lesions can present as cystic areas, which may be bleeding or necrotising (15, 16). Concordant with typical imaging manifestations, our case showed obvious parenchyma enhancement on arterial phase images and washout on delayed images on contrast-enhanced computed tomography (CT) and magnetic resonance imaging HCC.

Moreover, tumour serum markers, such as CEA, AFP, and carbohydrate antigen19-9 (CA19-9), and inflammatory indices (erythrocyte sedimentation rate and C-reactive protein), are generally normal. Recently, Li et al. reported a case of PHNET in a patient with chronic hepatitis C and highly elevated AFP (3).

A definite diagnosis of PHNET principally results from pathological examination and exclusion of other primary tumour sites (3, 4, 6). US- or CT-guided liver biopsy can be conducive to the preoperative diagnosis of uPHNET (6). A recent study reported that preoperative biopsy for NETs had a very good diagnostic rate and was confirmed by final histology, highlighting the reliability of biopsy for the diagnosis, grading, and differential diagnosis of NETs (17). Our patient underwent a US-guided core needle liver biopsy, and a pathological examination of the tissue suggested a grade 2 NET, consistent with the postoperative pathological results.

There were no significant differences in the pathological features of PHNETs and NETs in the digestive system. The tumour cells may be arranged in solid nests, ribbons, strips, and glandular tubes and may be accompanied by different degrees of blood sinus formation and interstitial fibrous tissue hyperplasia (18). The cytoplasm of the tumour is eosinophilic, and the nucleus is usually uniform in size, usually in small- or medium-sized cells. According to the proliferation activity assessed by mitotic count and Ki-67 proliferation index, NETs are classified as G1, G2, or G3 (19). The categorisation of PHNETs could be valuable for the assessment of the prognosis and malignancy of tumours (20). Not only that ChrA, Syno, and neuron-specific enolase (NSE) are considered important indicators for the diagnosis of PHNET (3, 8). Our present report exhibited positive expression of ChrA and Syno, and the patient was diagnosed with G2 NET.

However, a definitive diagnosis of a liver mass requires the exclusion of metastatic NET. The pancreas is a common primary site for NETs (21). Recently, a prospective study reported that endoscopic ultrasound (EUS) had a high negative predictive value for NETs and that tumours >20 mm had a poor prognosis (22). EUS is valuable in excluding the pancreas as the primary site. 68Ga-DOTATATE PET/CT has evolved as a promising tool for the evaluation of PHNET, which was performed to identify the primary site and rule out non-hepatic origins (23). In our case, the patient did not undergo pancreatic EUS but underwent PET-CT on our recommendation, which ruled out metastatic origins. Furthermore, a long-term follow-up was performed to confirm the diagnosis.

Currently, no evidence-based guidelines are available for the treatment of PHNETs. Complete surgical tumour resection with negative margins is considered the optimal scheme (3, 6, 8). A previous study reported that the 5-year survival rate after surgery was as high as 74%–78% (7). Moreover, surgical treatment exhibited a higher symptom remission rate than non-surgical treatment (20). However, for inoperable tumours, the optimal treatment modality continues to be debated, such as TACE, radiofrequency ablation, liver transplantation, and chemotherapy (8–11, 24). Stephen et al. reported that two patients with PHNET who underwent liver transplantation remained favourable, with no disease recurrence at 45 and 95 months (10). As a minimally invasive alternative therapeutic intervention, radiofrequency ablation has been reported as a potential treatment for NETs, which is relatively safe and effective for inducing necrosis and death of most NET cells (24, 25). However, further studies with more patients and longer follow-up periods are required to determine the prognosis of PHNETs. PHNETs are hypervascular and sensitive to ischaemia, similar to other primary hepatic hypervascularized tumours such as HCC (14). Thus, TACE can downsize tumours and convert uPHNET into resectable tumours. Park et al. reported that the overall survival of patients after TACE was identical to the mean survival of three patients with resectable PHNETs (8). Combination therapies for unresectable PHNETs may yield better outcomes than single-modality therapies. In the present case, the tumour size was reduced after TACE to improve the R0 resection rate and promote a potential survival benefit. The treatment resulted in a complete response, suggesting that pre-operative TACE could be an appropriate approach for shrinking the tumour.

However, our study had the main limitation of being a single case report with insufficient evidence to support the benefits of the treatment. The mechanisms of the biological behaviours of PHNETs and appropriate therapeutic strategies have not been clearly elucidated owing to the tumour rarity.

## Conclusion

In conclusion, the present case provides a potentially effective management strategy for uPHNET, which completely regressed with combined pre-operative TACE and surgery. The rare case showed that pre-operative TACE could be an appropriate approach to shrink refractory tumours. Further studies are required to confirm the effectiveness of this method.

## Data Availability Statement

The original contributions presented in the study are included in the article/supplementary material. Further inquiries can be directed to the corresponding authors.

## Ethics Statement

Written informed consent was obtained from the individual(s) for the publication of any potentially identifiable images or data included in this article.

## Author Contributions

XYZ, HRY, and HZL performed image acquisition and completed the manuscript. All authors contributed to the article and approved the submitted version.

## Funding

National Natural Science Foundation of China (30970839&31170957).

## Conflict of Interest

The authors declare that the research was conducted in the absence of any commercial or financial relationships that could be construed as a potential conflict of interest.

## Publisher’s Note

All claims expressed in this article are solely those of the authors and do not necessarily represent those of their affiliated organizations, or those of the publisher, the editors and the reviewers. Any product that may be evaluated in this article, or claim that may be made by its manufacturer, is not guaranteed or endorsed by the publisher.
